# The OR black box in urology: A survey on patient acceptance and confidence in AI‐assisted surgery

**DOI:** 10.1002/bco2.70288

**Published:** 2026-10-02

**Authors:** Julian Risch, Konrad Hügelmann, Leonhard Buck, Hans‐Christoph von Knobloch, Jakob Kohler, Oscar Weische, Reha‐Baris Incesu, Marie‐Luise Weiss, Neele Heckmann, Patricia Schließer, Jonas Jarczyk, Philipp Nuhn, Severin Rodler

**Affiliations:** ^1^ Department of Urology University Hospital of Schleswig‐Holstein Kiel Germany; ^2^ Kurt‐Semm Center for Laparoscopic and Robotic Surgery University Hospital of Schleswig Holstein Kiel Germany

**Keywords:** artificial intelligence, informed consent, intraoperative recording, operating room black box, patient acceptance, patient safety, surgical data recording, urology

## Abstract

**Objectives:**

This study aims to assess patient acceptance of intraoperative video recording with Operating Room (OR) black box technology, characterise consent conditions and use‐case preferences, and identify predictors of patient confidence in artificial intelligence (AI)‐assisted surgery in urological patients.

**Patients and Methods:**

This prospective cross‐sectional survey was conducted at the urology department of a university hospital from March 2024 to March 2025. Consecutive adults scheduled for elective procedures were enrolled (322 enrolled; 316 completed the binary consent item). A standardised questionnaire assessed consent to operative video storage and attitudes across four Likert‐scaled domains (29 items: consent conditions, use cases, data access and AI confidence). The primary outcome was confidence in AI‐assisted surgery for the full procedure. Analysis included Wilson 95% confidence intervals, between‐group comparisons and multivariable linear regression.

**Results:**

Of 316 patients (82.9% male, mean age 65.4 years), 304 (96.2%, 95% CI 93.5–97.8%) consented to operative video storage, with universal acceptance among oncological patients (181/181, 100%). Nonconsenters (*n* = 12) were younger, without oncological diagnosis and reported lower AI confidence. All 29 attitudinal items received majority endorsement. Patient confidence in AI‐assisted surgery was expressed by 55.2% for the full procedure, 63.5% for simple steps and 53.9% for complex steps (*p* < 0.001). Older patients expressed greater confidence (*r*ₛ = −0.21, *p* < 0.001). Age and three attitudinal composites independently predicted AI confidence (adjusted *R* ^2^ = 0.308), whereas demographic and clinical characteristics accounted for only 5.8% of variation.

**Conclusion:**

Urological patients broadly accept intraoperative recording and AI‐assisted surgery when transparent governance is ensured. Attitudinal factors outweigh demographic and clinical characteristics, supporting patient‐centred consent frameworks for OR black box implementation in urological practice.

## 1 INTRODUCTION

Intraoperative adverse events remain a major source of preventable surgical harm. A 2023 cohort study documented adverse events in 23.6% of hospital admissions, of which 22.7% were deemed preventable, with surgical and procedural events accounting for 30.4% of all harms.^1^ Despite decades of patient safety initiatives, systematic intraoperative performance monitoring remains largely absent from routine surgical practice. The operating room (OR) black box translates the aviation flight recorder concept to the surgical environment, capturing synchronised video, audio and physiological data throughout procedures without disrupting clinical workflows.^2^, ^3^ First‐year implementation data documented a median of 20 intraoperative errors per case and measurable skill differences between trainees and consultants.^3^ A 2025 systematic review confirmed value across patient safety, team behaviour, skills assessment and cost efficiency,^4^ with structured debriefing achieving near‐universal team endorsement.^5^ Artificial intelligence (AI) integration extends this potential further through automated performance metrics and real‐time decision support.

Urology is particularly suited to OR black box implementation: virtually all procedures are camera‐guided and every endourological intervention generates continuous video by default. In a German nationwide registry of 993 276 major urological procedures (2005–2021), minimally invasive approaches represented 26% of cases with exponentially growing robotic volume.^6^ Prospective OR black box analysis identified 138 clinically significant intraoperative adverse events in urological surgery, with more than half occurring during critical operative steps.^7^ However, implementation requires addressing ethical and legal barriers: under European data protection legislation, continuous recording necessitates transparent governance, defined data ownership and explicit informed consent.^8^ Patient acceptance is thus a necessary precondition for ethical implementation. Available evidence suggests that patients recognise the value of operative recording and wish to access their own data,^9^ yet existing studies are predominantly descriptive, lack prospective design and have not been conducted in urological populations.

To address this evidence gap, we conducted a prospective cross‐sectional survey at a urology department of a university hospital to assess patient acceptance of intraoperative video recording, characterise consent conditions and use cases, evaluate data access preferences and identify predictors of confidence in AI‐assisted surgery. To our knowledge, this represents the first prospective study of patient perspectives on OR black box implementation in a urological population, providing foundational evidence to inform patient‐centred consent frameworks for surgical data recording and AI‐assisted surgery.

## 2 PATIENTS AND METHODS

### 2.1 Study design and setting

This prospective cross‐sectional survey was conducted at the Department of Urology, University Medical Center Schleswig‐Holstein (UKSH), Campus Kiel, Germany, between March 2024 and March 2025. Ethics approval was granted by the institutional Ethics Committee (reference: D636/23). All participants provided written informed consent in accordance with the Declaration of Helsinki. This study is reported following the Strengthening the Reporting of Observational Studies in Epidemiology (STROBE) statement for cross‐sectional studies^10^ (Table S1).

### 2.2 Participants

Consecutive adults (≥18 years) scheduled for elective urological procedures were eligible, including oncological operations (radical prostatectomy, cystectomy, partial‐/nephrectomy) and endourological interventions (e.g. holmium laser enucleation of the prostate [HoLEP] and transurethral resection of the bladder [TURB]). Exclusion criteria were incapacity to consent and insufficient German‐language comprehension. Of 322 enrolled patients, 316 (98.1%) completed the binary consent item (primary analytical sample), 300 (93.2%) completed the primary outcome item and 243 (75.5%) had complete data for multivariable regression. A post hoc sensitivity analysis indicated that with n = 232 and five predictors, the regression had 80% power (*α* = 0.05, two‐tailed) to detect *f* ^2^ ≥ 0.057 (*R* ^2^ ≥ 0.054), well below the observed adjusted *R* ^2^ of 0.308.

### 2.3 Questionnaire development and structure

The questionnaire (‘The OR Black Box’, in German ‘Blackbox im OP’) was developed de novo by the study team specifically for this study. It was not adapted from, or validated against, a preexisting instrument. Questionnaires were distributed by the responsible surgeon during pre‐operative informed consent and collected by a dedicated study assistant, who entered responses into a standardised database. Responses were anonymous. No formal pilot was conducted. Section 1 captured sociodemographic and clinical data and binary consent to operative video storage (yes/no). Section 2 assessed attitudes across four domains on a 5‐point Likert scale (1 = *strongly agree*/*very confident*; 5 = *strongly disagree*/*very uncertain*): consent conditions (Q9, 8 items), acceptable use cases (Q10, 12 items), post‐operative data access preferences (Q11, nine items) and confidence in AI‐assisted surgery (Q12, three items). The full questionnaire with all item wordings is provided in Figure S1. Internal consistency was good to excellent across all scales (*α* = 0.84–0.95).

### 2.4 Statistical analysis

Continuous variables are summarised as mean ± SD and median (interquartile range [IQR]). Categorical variables as *n* (%). Proportions are reported with Wilson 95% confidence intervals (CI). Consent/nonconsent group differences were assessed by Mann–Whitney *U* test (Cohen's *d* with 95% CI) and Fisher's exact test. Bivariate associations between all 29 attitudinal items (Q9–Q11) and the primary outcome (Q12a; AI confidence, full procedure) were quantified by Spearman *r*ₛ. Domain composites were computed as item means (≥75% completion), with Q11i excluded owing to criterion overlap (*r*ₛ = 0.51). Associations of six sociodemographic predictors with individual items were tested across 174 predictor–item pairs (Bonferroni *α* = 0.000287). Differences across Q12a–c were examined by Friedman test with post hoc Wilcoxon signed‐rank tests (Bonferroni *α* = 0.017). Multivariable linear regression was estimated with Q12a as outcome and age, sex and three attitudinal composites as predictors (*n* = 232). A prespecified ordinal logistic regression served as sensitivity analysis. All tests were two‐tailed (*α* = 0.05). Analyses were performed in R 4.4 using the packages ordinal, MASS and rstatix.

## 3 RESULTS

### 3.1 Patient characteristics

In total, 322 patients were enrolled. Of these, 316 (98.1%) completed the binary consent item and form the analytical sample (Table 1). The cohort was predominantly male (82.9%), with a mean age of 65.4 ± 12.7 years. Urological tumour disease was the most common indication (57.3%), followed by urolithiasis (15.2%) and benign prostatic enlargement (12.0%). Sociodemographic and clinical characteristics did not differ significantly among consent groups for sex, visit urgency or health insurance (all *p* > 0.14), whereas age differed significantly (*p* = 0.006).

**TABLE 1:** Sociodemographic and clinical characteristics (*n* = 316).

Characteristic	Total (*n* = 316)	Consent YES (*n* = 304)	Consent NO (*n* = 12)	*p*
Age, years				*0.006*
Mean ± SD	65.4 ± 12.7	65.8 ± 12.4	52.8 ± 16.8	
Median (IQR)	67 (59–74)	67 (60–74)	52 (40–63)	
Not reported	7 (2.2%)	6 (2.0%)	1 (8.3%)	
Sex				*0.416*
Male	262 (82.9%)	253 (83.2%)	9 (75.0%)	
Female	50 (15.8%)	47 (15.5%)	3 (25.0%)	
Not reported	4 (1.3%)	4 (1.3%)	0 (0.0%)	
Educational attainment^1^				—
No or primary school	15 (4.7%)	13 (4.3%)	2 (16.7%)	
Lower secondary school	129 (40.8%)	129 (42.4%)	0 (0.0%)	
Upper secondary (Abitur)	51 (16.1%)	49 (16.1%)	2 (16.7%)	
Vocational training	47 (14.9%)	44 (14.5%)	3 (25.0%)	
University degree	68 (21.5%)	64 (21.1%)	4 (33.3%)	
Not reported	6 (1.9%)	5 (1.6%)	1 (8.3%)	
Primary indication^1^				—
Urological tumour disease	181 (57.3%)	181 (59.5%)	0 (0.0%)	
Urolithiasis	48 (15.2%)	45 (14.8%)	3 (25.0%)	
Benign prostatic enlargement	38 (12.0%)	35 (11.5%)	3 (25.0%)	
Other/unknown	44 (13.9%)	38 (12.5%)	6 (50.0%)	
Not reported	5 (1.6%)	5 (1.6%)	0 (0.0%)	
Visit urgency				*0.144*
Elective (≥4 weeks)	171 (54.1%)	167 (54.9%)	4 (33.3%)	
Urgent/emergency	138 (43.7%)	130 (42.8%)	8 (66.7%)	
Not reported	7 (2.2%)	7 (2.3%)	0 (0.0%)	
Health insurance				*1.000*
Statutory	239 (75.6%)	230 (75.7%)	9 (75.0%)	
Private	76 (24.1%)	73 (24.0%)	3 (25.0%)	
Not reported	1 (0.3%)	1 (0.3%)	0 (0.0%)	

*Note*: Percentages are based on total *n* per group. *p* = comparison of Consent YES (*n* = 304) versus Consent NO (*n* = 12); Mann–Whitney *U* test for age; Fisher's exact test for sex, visit urgency, and health insurance (computed on valid responses only, excluding not reported).^1^ Formal testing not performed: expected cell counts in the Consent NO group were <5 in the majority of categories; data are presented descriptively. IQR, interquartile range; SD, standard deviation.

### 3.2 Consent to operative video recording

Consent to operative video storage was granted by 304 of 316 patients (96.2%, 95% CI 93.5–97.8%). Consent was universal among oncological patients (181/181, 100%), compared with 93.8% for urolithiasis and 92.1% for benign prostatic enlargement. Nonconsenters (*n* = 12) were on average 13 years younger than consenters (52.8 vs. 65.8 years; *d* = 1.04, 95% CI 0.43–1.65), none carried an oncological diagnosis and they reported markedly lower AI confidence (mean Q12a 4.40 vs. 2.41; *d* = 1.56, 95% CI 0.92–2.21). All three differences reflect large effect sizes (*d* > 1.0). Given the small size of the nonconsenting group, between‐group comparisons should be interpreted with caution.

### 3.3 Consent conditions, use cases and data access preferences

All 29 items across Q9, Q10 and Q11 received majority endorsement (Figure 1). Within Q9 (consent conditions), agreement ranged from 95.0% (verbal and written information) to 51.4% (financial compensation for data transfer). Within Q10 (use cases), agreement ranged from 96.3% (medical documentation) to 80.3% (patient education by video). Within Q11 (data access), agreement ranged from 82.8% (documentation of complications) to 58.8% (endoscopic video only). Of 174 item‐level tests (29 items × 6 predictors), two survived Bonferroni correction: age was inversely associated with Q11i (*r*ₛ = −0.25, *p* < 0.001) and educational attainment differed across groups for Q11h (*p* < 0.001). No other sociodemographic predictor survived correction.

**FIGURE 1:**
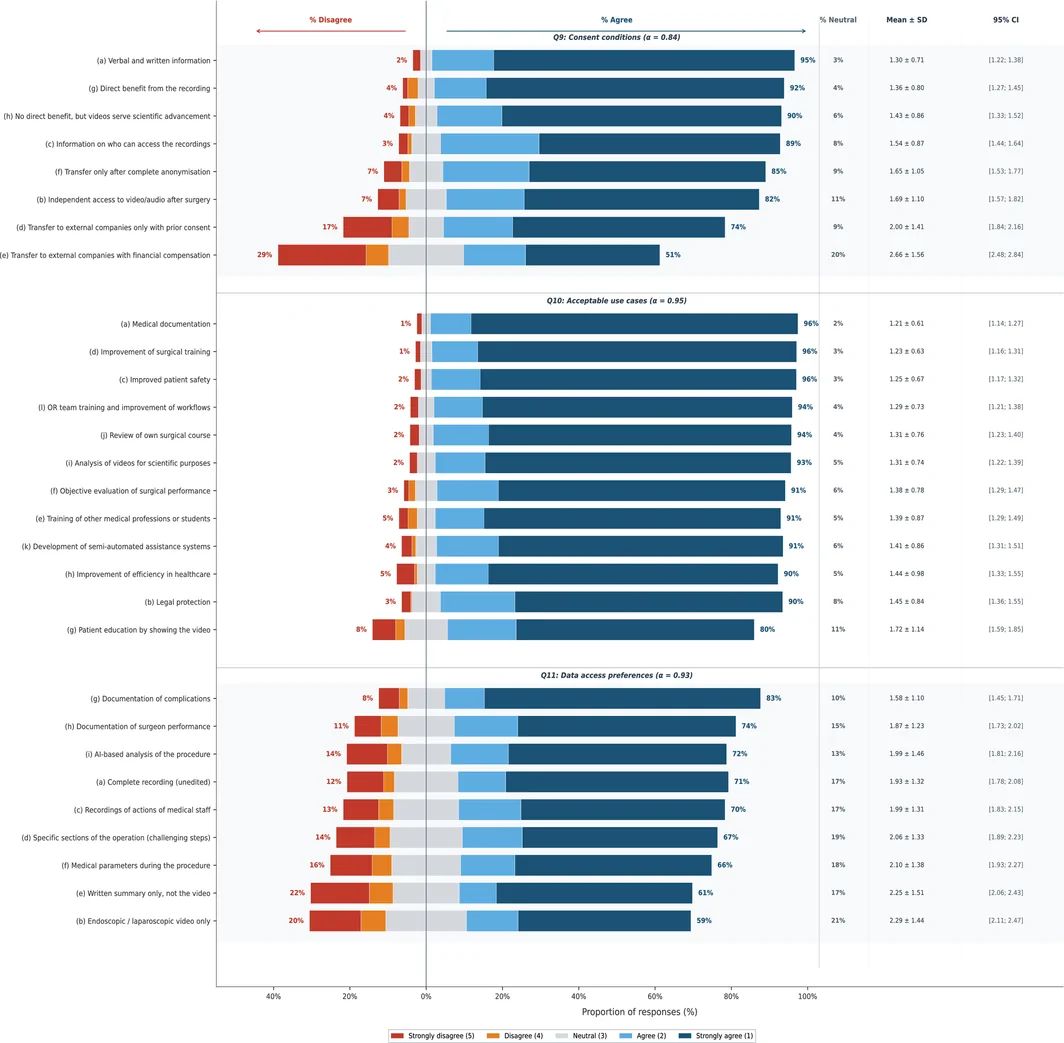
Patient endorsement of consent conditions (Q9, eight items; *α* = 0.84), acceptable use cases (Q10, 12 items; α = 0.95) and data access preferences (Q11, nine items; *α* = 0.93). Diverging stacked bars show the distribution of responses on a 5‐point Likert scale (1 = *strongly agree*, 5 = *strongly disagree*). Items are ranked by agreement within each domain. % agree = proportion scoring 1–2; % disagree = proportion scoring 4–5; % neutral = proportion scoring 3. Mean, *SD* and 95% confidence intervals of the mean are shown on the right. All 29 items achieved majority endorsement (>50% agreement). *α*, Cronbach's alpha; CI, confidence interval; SD, standard deviation.

### 3.4 Patient confidence in AI‐assisted surgery

Figure 2A illustrates patient responses across three AI‐assisted operative scenarios. A majority expressed confidence (Likert 1–2): 55.2% for the full procedure (Q12a; mean 2.48 ± 1.32; 95% CI 49.5%–60.8%), 63.5% for simple steps (Q12b; mean 2.31 ± 1.30; 95% CI 57.7%–68.9%) and 53.9% for complex steps (Q12c; mean 2.53 ± 1.36; 95% CI 48.1%–59.6%), indicating a task‐complexity gradient. A Friedman test confirmed a significant difference across scenarios (*χ* ^2^[2] = 54.39, *p* < 0.001, *n* = 281; Kendall's *W* = 0.097). Post hoc Wilcoxon signed‐rank tests (Bonferroni *α* = 0.017) showed higher confidence for simple steps compared with both the full procedure (*p* < 0.001) and complex steps (*p* < 0.001), whereas the full procedure and complex steps did not differ (*p* = 0.489). An inverse age gradient was observed (Figure 2B): mean Q12a scores decreased across age quartiles from 2.91 (<60 years) to 2.58 (60–67 years), 2.16 (68–74 years) and 2.11 (≥75 years), indicating greater confidence among older patients (Spearman *r*ₛ = −0.21, *p* < 0.001). This association was present in both oncological (*r*ₛ = −0.20, *p* = 0.009, *n* = 171) and non‐oncological subgroups (*r*ₛ = −0.27, *p* = 0.004, *n* = 115). An age × diagnosis interaction term was not significant (*p* = 0.984).

**FIGURE 2:**
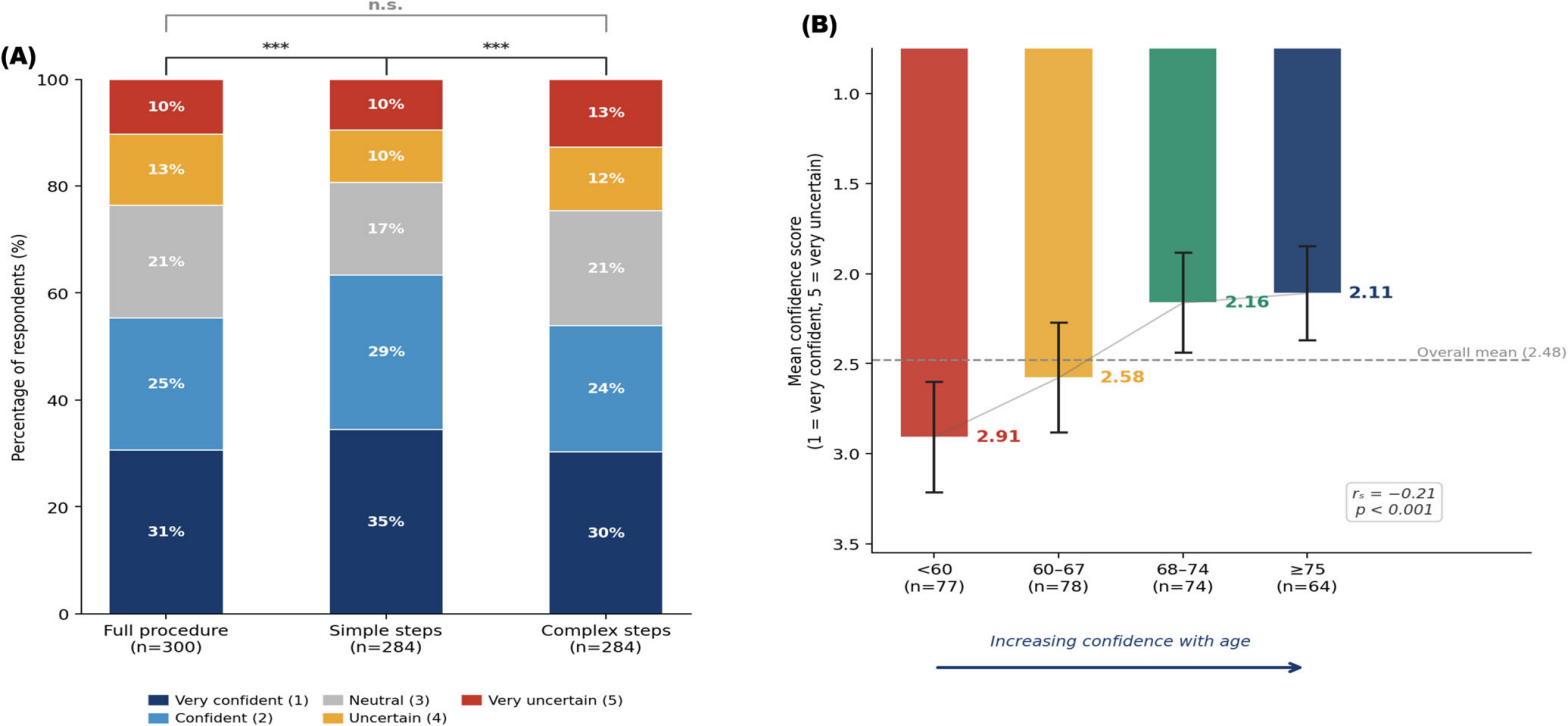
Patient confidence in AI‐assisted surgery (Q12). (A) Response distribution for Q12 across three operative scenarios (full procedure, simple steps and complex steps) on a 5‐point Likert scale (1 = *very confident*, 5 = *very uncertain*). Significance brackets indicate post hoc Wilcoxon signed‐rank test results with Bonferroni correction (*α* = 0.017); *** *p* < 0.001; n.s. = not significant. (B) Mean Q12a score (full procedure) by age quartile (*n* = 293; seven patients excluded owing to missing age data): error bars represent 95% confidence intervals. Lower scores indicate greater confidence. Dashed line indicates the overall mean (2.48). Spearman *r* ₛ = −0.21, *p* < 0.001 (age as continuous variable). CI, confidence interval; Q12, questionnaire section 12.

### 3.5 Predictors of AI‐assisted surgery confidence

All 29 items (Q9–Q11) showed significant positive associations with AI confidence (*r*ₛ = 0.31–0.53, all *p* < 0.001; Table S2). All three attitudinal composites were more strongly associated with AI confidence than any sociodemographic variable: consent conditions *r*ₛ = 0.47, use intentions *r*ₛ = 0.43, data access *r*ₛ = 0.45 (all *p* < 0.001). In multivariable regression (*n* = 232, adjusted *R* ^2^ = 0.308, *F*[5226] = 21.45, *p* < 0.001), age (β* = −0.151, *p* = 0.009), consent conditions composite (β* = 0.190, *p* = 0.027), use intentions composite (β* = 0.222, *p* = 0.009) and data access composite (β* = 0.201, *p* = 0.002) were independent predictors (Table 2). Sex was not significant (*p* = 0.785). All VIF < 3. A model restricted to sociodemographic and clinical predictors (age, sex, diagnostic group, visit urgency, insurance) explained only 5.8% of variance (adjusted *R* ^2^ = 0.058, *n* = 278). Ordinal logistic regression yielded consistent results (Table S3).

**TABLE 2:** Multivariable linear regression: predictors of patient confidence in AI‐assisted surgery (full procedure; *n* = 232).

Predictor	*b*	*SE*	β*	95% CI	*p*‐value
Age (years)	−0.016	0.006	−0.151	[−0.027; −0.004]	*0.009*
Sex (male vs. female)	−0.048	0.176	−0.015	[−0.393; 0.297]	*0.785*
Consent conditions (Q9; eight items)	0.345	0.155	0.190	[0.041; 0.649]	*0.027*
Use intentions (Q10; 12 items)	0.444	0.168	0.222	[0.115; 0.773]	*0.009*
Data access (Q11; eight items)	0.246	0.078	0.201	[0.094; 0.399]	*0.002*
Intercept	1.809	0.461	—	[0.900; 2.718]	*<0.001*
Model fit					
*n* (complete cases)	232				
Adjusted *R* ^2^	0.308				
*F*(5, 226)	21.45			*p < 0.001*	

*Note*: *b*, unstandardised OLS coefficient; β*, standardised coefficient (SD units); SE, standard error; CI, confidence interval; *p*, two‐tailed *p*‐value from *t*‐test (*df* = 226). *F*(5, 226), *F*‐statistic with five predictor degrees of freedom and 226 residual degrees of freedom (*n* − k − 1 = 232–5 − 1). Q11 composite excludes Q11i (AI‐based analysis of the procedure) owing to criterion contamination (*r*ₛ = 0.54 with Q12a). All VIF < 3 (max 2.42). Results were consistent in a prespecified sensitivity analysis using ordinal logistic regression (Table S2).

## 4 DISCUSSION

In this prospective cross‐sectional survey of 316 urological patients at a university hospital, four principal findings emerged. First, consent to intraoperative video recording was near‐universal (96.2%), with 100% acceptance among oncological patients. Second, all 29 attitudinal items achieved majority agreement, with informed consent, anonymisation and direct patient benefit ranking highest. Third, more than half of patients expressed confidence in AI‐assisted surgery, with a task‐complexity gradient favouring simple over complex steps. Fourth, AI confidence was independently predicted by age and all three attitudinal composites but not by sex or diagnostic group, explaining 30.8% of outcome variance.

The consent rate of 96.2% is consistent with a Danish OR black box study reporting 98% acceptance (*n* = 306)^11^ and a French nationwide survey reporting 84.8% support,^12^ confirming high acceptance when transparent governance is ensured.^13^, ^14^ Universal acceptance among oncological patients may reflect that patients facing life‐threatening diagnoses may perceive video documentation as a quality safeguard, consistent with the observation that disease severity positively modulates technology acceptance in surgical settings.^15^ Nonconsenters (*n* = 12) were younger (mean 52.8 vs. 65.8 years; *d* = 1.04), carried no oncological diagnosis and reported lower AI confidence (Q12a 4.40 vs. 2.41; *d* = 1.56), suggesting that technology scepticism represents a coherent attitudinal stance rather than isolated reluctance towards recording. Given the small size of this subgroup, these observations should be interpreted cautiously. Future studies should prospectively capture patient‐reported refusal reasons. The high agreement across consent conditions (Q9) and use cases (Q10) indicates that patients expect transparent governance rather than categorically opposing recording. The strongest prerequisites, verbal and written information (95.0%), independent video access (82.0%) and transfer only after anonymisation (84.5%), closely mirror core General Data Protection Regulation (GDPR) requirements.^8^ This preference is consistent with a UK patient survey (*n* = 408) in which 77.9% were willing to share health data with the NHS but only 26.4% with commercial companies.^16^ Notably, scientific value without personal benefit (Q9h, 90.3%) also achieved near‐universal agreement, indicating widespread altruistic motivation. Among use cases, medical documentation (96.3%), patient safety (95.6%) and surgical training (95.7%) ranked highest, reaffirming the safety rationale for OR black box systems.^3^, ^4^ The minimal influence of sociodemographic variables on attitudinal items suggests that acceptance is broadly distributed across patient populations.

More than half of patients expressed confidence in AI‐assisted surgery (55.2%, 95% CI 49.5–60.8%), with higher confidence for simple steps (63.5%) than complex steps (53.9%; *p* < 0.001). This closely mirrors the 57.6% favourable attitude in the multinational COMFORT study (*n* = 13 806)^17^ and aligns with a US survey demonstrating that patient comfort with AI varies by clinical context.^18^ The task‐complexity gradient is further supported by an oncology survey (*n* = 330) in which comfort ranged from 80.2% for screening to 64.8% for treatment planning.^19^ Given that patients accept AI more readily for simple than complex tasks, clinicians introducing AI systems should emphasise their assistive rather than autonomous role.^20^ The inverse age gradient, with older patients expressing greater confidence in AI‐assisted surgical procedures, was unexpected. The stratified analysis reported earlier shows that this persists independently of oncological diagnosis and is not attributable to diagnostic confounding. Possible explanations include greater cumulative health‐care experience and higher baseline trust in the surgical team among older patients, as well as generational differences in deference to physician‐endorsed technology. In contrast, Rodler et al. found that technology affinity rather than age predicted AI trust in 466 prostate cancer patients.^21^ As technology affinity was not measured in our study, the observed age gradient may partly reflect unmeasured attitudinal differences between age groups. The multivariable model explained 30.8% of AI confidence variance (adjusted *R* ^2^ = 0.308), with all three attitudinal composites as independent predictors alongside age. Sociodemographic and clinical predictors alone explained only 5.8% of variance, underscoring that attitudinal rather than demographic factors drive technology acceptance. This is consistent with technology acceptance frameworks in which trust is a primary determinant of adoption intent. A recent meta‐analysis identified trust as the single strongest predictor of intention to use AI‐enabled clinical decision support (*r* = 0.73).^22^ The independent contribution of all three attitudinal domains suggests that trust in data governance, perceived usefulness of recording and desire for personal data access each play a distinct role in shaping AI confidence. However, the 69.2% unexplained variance implies that important predictors remain unmeasured, including health literacy, prior technology experience and personality traits, such as openness to innovation.^17^, ^23^ Patient health and technology literacy may be a particularly relevant unmeasured factor, as patients with lower literacy may be less able to weigh the risks and benefits of novel recording and AI‐assistance technologies, potentially influencing both consent behaviour and confidence ratings. Disparities in patients' understanding of and comfort with technology‐based care have similarly been reported for other technology‐enabled urological services.^24^ Sex was not significant, possibly reflecting the predominantly male cohort. The consistency of linear and ordinal logistic regression results supports the robustness of these findings. Several limitations should be acknowledged. This study enrolled a predominantly male (82.9%), oncologically enriched (57.3%) cohort at a single university hospital, which may limit generalisability. The cross‐sectional design precludes causal inference and attitudes may change following the actual exposure to AI‐assisted surgery. The questionnaire was distributed by the operating surgeon during pre‐operative consent, which may have inflated acceptance rates through social desirability bias. The questionnaire itself was developed de novo by the study team and was not adapted from, or benchmarked against, an externally validated instrument, which may limit comparability with previously validated scales. The attitudinal composites were developed de novo without external validation and may partly reflect a common latent construct such as general technology openness rather than distinct causal drivers. No formal pilot study was conducted, and reasons for nonconsent were not captured. The regression included only 232 of 316 patients with complete responses. Comparison of included and excluded patients showed no significant differences in baseline characteristics or primary outcome (all *p* > 0.15), suggesting that missing data did not introduce systematic bias.

## 5 CONCLUSION

Urological patients demonstrate high acceptance of intraoperative video recording and broadly supportive attitudes toward AI‐assisted surgery, provided that clear governance frameworks, particularly informed consent, anonymisation and independent data access, are in place. Acceptance was not uniform: oncological patients consented universally; older patients expressed greater AI confidence and attitudinal openness across consent conditions; use cases and data access were the strongest independent predictor of AI confidence, outweighing all sociodemographic factors. These findings provide an empirical basis for patient‐centred consent frameworks and support the ethical implementation of OR black box systems in urological practice. Future multi‐centre studies should include qualitative exploration of refusal reasons and validation of the attitudinal scales in diverse surgical populations.

## AUTHOR CONTRIBUTIONS

*Study concept and design*: Risch, Rodler, Hügelmann. *Acquisition of data*: Risch, Hügelmann, Buck, Kohler, Weische, Incesu, Weiss, Heckmann, Schließer, von Knobloch. *Analysis and interpretation of data*: Risch, Rodler. *Drafting of the manuscript*: Risch, Rodler. *Critical revision of the manuscript for important intellectual content*: Jarczyk, Nuhn, Rodler. *Statistical analysis*: Risch, Rodler. *Administrative, technical, or material support*: Risch, Rodler. *Supervision*: Nuhn, Rodler. Severin Rodler had full access to all the data in the study and takes responsibility for the integrity of the data and the accuracy of the data analysis.

## CONFLICT OF INTEREST STATEMENT

The authors have no conflict of interest to disclose.

## Supporting information


**Figure S1.** Questionnaire: “Blackbox im OP” — English translation and Original German questionnaire.
**Table S1.** STROBE Statement ‐ Checklist of items that should be included in reports of cross‐sectional studies.
**Table S2.** Spearman rank correlations of all Q9–Q11 items with AI confidence score (Q12a).
**Table S3.** Multivariable regression results: OLS (primary model) and ordinal logistic regression (sensitivity analysis).

## Data Availability

The data that support the findings of this study are available from the corresponding author upon reasonable request.
