# Supplementary material for: The OR black box in urology: A survey on patient acceptance and confidence in AI‐assisted surgery
Source: BJUI Compass. 2026 Oct 2;7(10):e70288. doi: 10.1002/bco2.70288 (PMC13631720; doi:10.1002/bco2.70288)
Supplement: Supplementary file 1 [file BCO2-7-e70288-s001.docx]

**Supplementary Figure 1.** Questionnaire: "Blackbox im OP" — English translation and Original German questionnaire

**Supplementary Table 1.** STROBE Statement - Checklist of items that should be included in reports of cross-sectional studies

|  | Item No | Recommendation | Location in manuscript |
| --- | --- | --- | --- |
| **Title and abstract** | 1 | (*a*) Indicate the study’s design with a commonly used ter in the title or the abstract | *Abstract, Methods sentence: 'Prospective cross-sectional survey at a university hospital urology department'* |
|  |  | (*b*) Provide in the abstract an informative and balanced summary of what was done and what was found | *Abstract (full): Objectives / Subjects-Patients and Methods / Results / Conclusion* |
| Introduction | | | |
| Background/rationale | 2 | Explain the scientific background and rationale for the investigation being reported | *Introduction, paragraphs 1 and 2* |
| Objectives | 3 | State specific objectives, including any prespecified hypotheses | *Introduction, paragraph 3 (last paragraph); Abstract, Objectives* |
| Methods | | | |
| Study design | 4 | Present key elements of study design early in the paper | *Methods, 'Study Design and Setting'; Abstract, Methods* |
| Setting | 5 | Describe the setting, locations, and relevant dates, including periods of recruitment, exposure, follow-up, and data collection | *Methods, 'Study Design and Setting' (Department of Urology, UKSH Campus Kiel; March 2024 – March 2025)* |
| Participants | 6 | (*a*) Give the eligibility criteria, and the sources and methods of selection of participants | *Methods, 'Participants' (consecutive adults ≥18 years scheduled for elective urological procedures; oncological and endourological)* |
| Variables | 7 | Clearly define all outcomes, exposures, predictors, potential confounders, and effect modifiers. Give diagnostic criteria, if applicable | *Methods, 'Questionnaire Development and Structure' (29 items; four Likert-scaled domains: consent conditions, use cases, data access, AI confidence)* |
| Data sources/ measurement | 8* | For each variable of interest, give sources of data and details of methods of assessment (measurement). Describe comparability of assessment methods if there is more than one group | *Methods, 'Questionnaire Development and Structure' (paper questionnaire distributed by responsible surgeon during pre-operative informed consent; standardised database entry by dedicated study assistant)* |
| Bias | 9 | Describe any efforts to address potential sources of bias | *Methods, 'Participants' (consecutive enrolment); Discussion, Limitations (social desirability, single-centre, non-validated de novo scales)* |
| Study size | 10 | Explain how the study size was arrived at | *Methods, 'Participants' (consecutive enrolment of all eligible patients across a 12-month period); Results (322 enrolled, 316 analysed)* |
| Quantitative variables | 11 | Explain how quantitative variables were handled in the analyses. If applicable, describe which groupings were chosen and why | *Methods, 'Statistical Analysis' (continuous: mean ± SD and median IQR; categorical: n (%); 5-point Likert scales analysed as ordinal with cut-points 1–2 for confidence)* |
| Statistical methods | 12 | (*a*) Describe all statistical methods, including those used to control for confounding | *Methods, 'Statistical Analysis' (Wilson 95% CIs; chi-squared / Fisher exact / Mann-Whitney U for group comparisons; Spearman correlation; multivariable linear regression)* |
|  |  | (*b*) Describe any methods used to examine subgroups and interactions | *Methods, 'Statistical Analysis'; Results, Predictors of AI confidence (subgroup: oncological vs non-oncological; attitudinal composites)* |
|  |  | (*c*) Explain how missing data were addressed | *Results, Patient Characteristics (322 enrolled, 316 completed binary consent item — 6 excluded for missing primary outcome; complete-case analysis)* |
|  |  | (*d*) If applicable, describe analytical methods taking account of sampling strategy | *N/A* |
|  |  | (*e*) Describe any sensitivity analyses | *N/A* |
| Results | | | |
| Participants | 13* | (a) Report numbers of individuals at each stage of study—eg numbers potentially eligible, examined for eligibility, confirmed eligible, included in the study, completing follow-up, and analysed | *Results, 'Patient Characteristics' (322 enrolled, 316 completed the binary consent item and form the analytical sample, 98.1%)* |
|  |  | (b) Give reasons for non-participation at each stage | *Results, 'Patient Characteristics' (6 patients excluded — missing primary outcome)* |
|  |  | (c) Consider use of a flow diagram | *Not included (single inclusion stage; numbers reported in text); Supplementary Material available if required* |
| Descriptive data | 14* | (a) Give characteristics of study participants (eg demographic, clinical, social) and information on exposures and potential confounders | *Results, 'Patient Characteristics'; Table 1 (age, sex, diagnosis, scheduled procedure, oncological vs non-oncological)* |
|  |  | (b) Indicate number of participants with missing data for each variable of interest | *Table 1 and Results text (per-item denominators reported where missing data occurred)* |
| Outcome data | 15* | Report numbers of outcome events or summary measures | *Results, 'Consent to Operative Video Recording' (304/316 consented, 96.2%, 95% CI 93.5–97.8%); 'Patient Confidence in AI-Assisted Surgery' (55.2% / 63.5% / 53.9% across procedure complexity)* |
| Main results | 16 | (*a*) Give unadjusted estimates and, if applicable, confounder-adjusted estimates and their precision (eg, 95% confidence interval). Make clear which confounders were adjusted for and why they were included | *Results, 'Predictors of AI-Assisted Surgery Confidence' (Spearman correlations rₛ = 0.31–0.53 unadjusted; multivariable linear regression adjusted R² = 0.308 with age and three attitudinal composites)* |
|  |  | (*b*) Report category boundaries when continuous variables were categorized | *Likert 1–2 'confidence' definition stated in Methods/Statistical Analysis* |
|  |  | (*c*) If relevant, consider translating estimates of relative risk into absolute risk for a meaningful time period | *N/A* |
| Other analyses | 17 | Report other analyses done—eg analyses of subgroups and interactions, and sensitivity analyses | *Results, 'Predictors of AI-Assisted Surgery Confidence' (subgroup: oncological vs non-oncological)* |
| Discussion | | | |
| Key results | 18 | Summarise key results with reference to study objectives | *Discussion, paragraph 1 (four principal findings)* |
| Limitations | 19 | Discuss limitations of the study, taking into account sources of potential bias or imprecision. Discuss both direction and magnitude of any potential bias | *Discussion, Limitations subsection (single-centre, predominantly male oncological cohort, cross-sectional design, social desirability bias, non-validated de novo scales)* |
| Interpretation | 20 | Give a cautious overall interpretation of results considering objectives, limitations, multiplicity of analyses, results from similar studies, and other relevant evidence | *Discussion, paragraphs 2–4 (Danish and French comparator studies, ethical and legal context, AI-confidence interpretation)* |
| Generalisability | 21 | Discuss the generalisability (external validity) of the study results | *Discussion, Limitations subsection (single-centre, predominantly oncological cohort, generalisability to general urological population discussed)* |
| Other information | | | |
| Funding | 22 | Give the source of funding and the role of the funders for the present study and, if applicable, for the original study on which the present article is based | *End of manuscript: 'Funding/Support and Role of the Sponsor: None.'* |

*Give information separately for exposed and unexposed groups.

**Note:** An Explanation and Elaboration article discusses each checklist item and gives methodological background and published examples of transparent reporting. The STROBE checklist is best used in conjunction with this article (freely available on the Web sites of PLoS Medicine at http://www.plosmedicine.org/, Annals of Internal Medicine at http://www.annals.org/, and Epidemiology at http://www.epidem.com/). Information on the STROBE Initiative is available at www.strobe-statement.org.

**Supplementary Table 2.** Spearman rank correlations of all Q9–Q11 items with AI confidence score (Q12a).

| **Item** | **Description** | **rₛ** | **n** | **p** |
| --- | --- | --- | --- | --- |
|  | **Q9 — Consent conditions (8 items; composite α = 0.84)** |  |  |  |
| Q9a | Verbal and written information | 0.309 | 287 | *<0.001* |
| Q9b | Independent access to video/audio after surgery | 0.317 | 270 | *<0.001* |
| Q9c | Provision of information on who can access recordings | 0.327 | 277 | *<0.001* |
| Q9d | Transfer to external companies only with prior consent | 0.340 | 282 | *<0.001* |
| Q9e | Transfer with financial compensation | 0.345 | 274 | *<0.001* |
| Q9f | Transfer only after complete anonymisation | 0.348 | 282 | *<0.001* |
| Q9g | I have a direct benefit from the recording | 0.368 | 286 | *<0.001* |
| Q9h | I have no direct benefit, but videos serve science | 0.346 | 286 | *<0.001* |
|  | **Q10 — Acceptable use cases (12 items; composite α = 0.95)** |  |  |  |
| Q10a | Medical documentation | 0.378 | 288 | *<0.001* |
| Q10b | Legal protection | 0.374 | 283 | *<0.001* |
| Q10c | Improved patient safety | 0.341 | 287 | *<0.001* |
| Q10d | Improvement of surgical training | 0.399 | 289 | *<0.001* |
| Q10e | Training of other medical professions or students | 0.380 | 287 | *<0.001* |
| Q10f | Objective evaluation of surgical performance | 0.396 | 288 | *<0.001* |
| Q10g | Patient education by showing video | 0.402 | 288 | *<0.001* |
| Q10h | Improvement of efficiency in healthcare | 0.361 | 286 | *<0.001* |
| Q10i | Analysis of videos for scientific purposes | 0.308 | 290 | *<0.001* |
| Q10j | Analysis of videos to review own surgical course | 0.367 | 289 | *<0.001* |
| Q10k | Semi-automated surgical assistance systems | 0.451 | 282 | *<0.001* |
| Q10l | OR team training and workflow improvement | 0.343 | 283 | *<0.001* |
|  | **Q11 — Data access preferences (9 items; composite α = 0.93, excl. Q11i)** |  |  |  |
| Q11a | Complete recording (unedited) | 0.410 | 273 | *<0.001* |
| Q11b | Endoscopic / laparoscopic video only | 0.330 | 240 | *<0.001* |
| Q11c | Recordings of actions of medical staff | 0.357 | 252 | *<0.001* |
| Q11d | Specific sections (challenging steps) | 0.348 | 242 | *<0.001* |
| Q11e | Written summary only, not the video | 0.319 | 253 | *<0.001* |
| Q11f | Medical parameters during procedure | 0.446 | 249 | *<0.001* |
| Q11g | Documentation of complications | 0.339 | 274 | *<0.001* |
| Q11h | Documentation of surgeon performance | 0.373 | 269 | *<0.001* |
| Q11i | AI-based analysis of the procedure ^n^ | 0.534 | 269 | *<0.001* |

rₛ = Spearman rank correlation coefficient. All correlations are with Q12a (AI confidence for the full procedure; 1 = very confident, 5 = very uncertain). Domain composites used in multivariable regression are shown with item count and Cronbach’s α. Q11i excluded from the data access composite owing to criterion contamination (rₛ = 0.53 with Q12a). All p < 0.001.

**Supplementary Table 3.** Multivariable regression results: OLS (primary model) and ordinal logistic regression (sensitivity analysis).

|  | **OLS (primary model)** | | | | | **Ordinal logistic regression (sensitivity)** | | |
| --- | --- | --- | --- | --- | --- | --- | --- | --- |
| **Predictor** | **b** | **SE** | **β*** | **95% CI** | **p** | **OR** | **95% CI** | **p** |
| Age | -0.016 | 0.006 | -0.151 | [-0.027; -0.004] | *0.009* | 0.977 | [0.959; 0.997] | *0.021* |
| Sex (male) | -0.048 | 0.176 | -0.015 | [-0.393; 0.297] | *0.785* | 0.850 | [0.447; 1.617] | *0.621* |
| Q9 — Consent conditions | 0.345 | 0.155 | 0.190 | [0.041; 0.649] | *0.027* | 2.132 | [1.277; 3.559] | *0.004* |
| Q10 — Use intentions | 0.444 | 0.168 | 0.222 | [0.115; 0.773] | *0.009* | 2.430 | [1.365; 4.326] | *0.003* |
| Q11 — Data access | 0.246 | 0.078 | 0.201 | [0.094; 0.399] | *0.002* | 1.469 | [1.115; 1.936] | *0.006* |
| *Model fit: n = 232; OLS Adj. R² = 0.308; F(5,226) = 21.45, p < 0.001 \| Ordinal LR: AIC = 637.0* | | | | | | | | |

b = unstandardized OLS coefficient; β* = standardized coefficient; SE = standard error; OR = odds ratio from ordinal logistic regression (proportional odds model); 95% CI = 95% confidence interval. Outcome: Q12a (AI confidence for the full procedure; 1 = very confident, 5 = very uncertain). Q11 composite excludes Q11i owing to criterion contamination (rₛ = 0.54 with Q12a). All VIF < 3 (max 2.42). OR > 1 indicates higher odds of being in a less confident category (higher score).
